# Visual similarity effects on masked priming

**DOI:** 10.3758/s13421-013-0388-4

**Published:** 2013-12-17

**Authors:** Sachiko Kinoshita, Serje Robidoux, Luke Mills, Dennis Norris

**Affiliations:** 1 ARC Centre of Excellence in Cognition and its Disorders (CCD), Macquarie University, Sydney, NSW 2109 Australia; 2 Department of Psychology, Macquarie University, Sydney, NSW Australia; 3 MRC Cognition and Brain Sciences Unit, Cambridge, UK

**Keywords:** Word recognition, Lexical processing, Repetition priming

## Abstract

We investigated the role of the visual similarity of masked primes to targets in a lexical decision experiment. In the primes, some letters in the target (e.g., A in ABANDON) had either visually similar letters (e.g., H), dissimilar letters (D), visually similar digits (4), or dissimilar digits (6) substituted for them. The similarities of the digits and letters to the base letter were equated and verified in a two-alternative forced choice (2AFC) perceptual identification task. Using targets presented in lowercase (e.g., abandon) and primes presented in uppercase, visually similar *digit* primes (e.g., 484NDON) produced more priming than did visually dissimilar digit primes (676NDON), but little difference was found between the visually similar and dissimilar *letter* primes (HRHNDON vs. DWDNDON). These results were explained in terms of task-driven competition between the target letter and the visually similar letter.

People are remarkably efficient at recognizing letters, despite the variability in surface form—for instance, A, A, and a are all readily recognized as instances of the letter “a,” across their variations in size, font, and case. Consistent with this observation, most current visual word recognition models assume that the letter representations subserving word recognition are “abstract letter identities.” This assumption is shared both by models adopting the interactive-activation framework (originally put forward by McClelland & Rumelhart, 1981)—such as the dual-route cascaded (DRC) model (Coltheart, Rastle, Perry, Langdon, & Ziegler, 2001), the multiple read-out model (MROM; Grainger & Jacobs, 1996), the spatial-coding model (SCM; Davis, 2010), and various “open-bigram” models (e.g., SERIOL: Whitney, 2001, 2008; or the parallel open-bigram model: Grainger & Van Heuven, 2004)—and those that eschew the activation metaphor and instead regard visual word recognition as a Bayesian inference-making process—such as the Bayesian Reader (Norris, 2006), and its successors, the noisy slot model (Norris, Kinoshita, & van Casteren, 2010) and the noisy-channel model (Norris & Kinoshita, 2012). In the latter frameworks, the reader is characterized as making an optimal Bayesian inference in mapping noisy perceptual evidence onto a word that best matches the input.

The topic investigated in the present article concerns the role of the visual similarity of letters in the early stages of visual word recognition in skilled readers. We first present a brief review of the masked-priming literature that seems to suggest two conflicting views, with one group of studies suggesting that the visual similarity of the prime and target letters has no bearing on priming, and the other suggesting that it does. We will argue, however, that the conflict is more apparent than real, and that these studies do not address the question of whether the visual similarity between letters matters. We then present an experiment, testing whether a prime containing letter substitutions with visually similar letters produces more priming than a prime containing visually dissimilar letter substitutions.

As is apparent from studies that have examined the identification of isolated letters (for a recent review, see Mueller & Weidemann, 2012), letters such as E and F, and C and O, are visually similar and confusable with each other. However, in current models of visual word recognition, the letter representations that feed into lexical representations are assumed to be abstract, and the visual similarity between letters plays a minimal role in word recognition itself. This is consistent with studies based on masked priming that have shown that the visual similarity of the letters in the prime and the target has no impact on the size of the identity priming. In the masked-priming procedure developed by Forster and Davis (1984), which has been adopted as the standard in visual word recognition studies using the Latin alphabet, the prime is presented in lowercase letters and the target in uppercase, so as to avoid physical overlap (e.g., table–TABLE). Capitalizing on this, Bowers, Vigliocco, and Haan (1998) elegantly demonstrated that the letter representations supporting word recognition are abstract. They compared the sizes of the identity-priming effects for words consisting of letters that are visually similar in uppercase and lowercase (e.g., kiss–KISS) and words consisting of letters that are visually dissimilar across cases (e.g., edge–EDGE). In both a lexical decision task and a noun–verb decision task, visual similarity had little effect on the size of priming, leading Bowers et al. to conclude that masked priming in word recognition is based on abstract letter identities.^1^ Perea, Abu Mallouh, and Carreiras (2013) recently extended this finding to Arabic. Although Arabic does not have uppercase and lowercase letters, it has an intricate system of allography. Arabic is written cursively from right to left and has 28 letters. Two variables interact to determine which allograph is used for a given letter. These are the position of the letter in the word—initial, medial, or final—and the presence or absence of a ligature (i.e., whether the letter is connected) to the preceding letter. Like Hebrew, another Semitic language, words in Arabic can be decomposed into a three-letter consonantal root that conveys the basic meaning (e.g., *ktb* for “marking/writing”) and a phonological word pattern consisting of a sequence of vowels (or a sequence of vowels and consonants), which is embedded in the root. Perea et al. (2013) created primes by substituting a word pattern letter with either a letter that kept the same ligation pattern as in the target word (visually similar) or one that altered the ligation pattern (visually dissimilar). The magnitude of priming did not differ between the “same-ligation” and “different-ligation” conditions, indicating that the visual similarity of allographs did not influence masked priming.

In contrast to these studies, studies using “leet” primes—letter strings that contain digits (or symbols) resembling letters embedded in a letter string: for example, M4T3R14L for MATERIAL—have shown that priming is determined by the visual similarity between the prime and target. Perea, Duñabeitia, and Carreiras (2008; see also Carreiras, Duñabeitia, & Perea, 2007) contrasted leet primes containing digits that were visually similar to the replaced letter (the “related leet” primes; e.g., M4T3R14L) and primes containing digits that did not resemble the replaced letter (the “control leet” primes; e.g., M5T6R28L), and found that whereas the related leet primes facilitated the recognition of the target almost as much as the identity primes (e.g., MATERIAL), the control leet primes produced little more facilitation than did control letter primes (e.g., MOTURUOL). These results indicate that the visual similarity between the digit and the replaced letter was critical in producing priming.

On the surface, these two sets of findings appear to suggest a contradiction: On the one hand, studies manipulating the visual similarity of allographs (uppercase and lowercase letters in English, or ligation-dependent allographs in Arabic) revealed no role for visual similarity in modulating priming; on the other hand, studies using leet primes showed that the visual similarity of the digit/symbol is the key factor responsible for priming. We suggest, however, that the contradiction is more apparent than real. The two sets of studies differ in the ways that visual similarity was manipulated. Specifically, in the allograph studies, the manipulation concerned the visual similarity between different instances of the *same* letter identity (A, a, A, etc.) in the prime and target—that is, between tokens that mapped onto the same letter type. In these cases, the form of the letter does not matter, indicating that visual word recognition is based on abstract letter identities.

In contrast, in the leet-priming studies, the visual-similarity manipulation concerned similarity between an input and a letter type. To the extent that *different letter identities* are defined by their shape, for a visual input (e.g., the digit 4 or 8, or the symbol €) to be taken as an instance of a particular letter (e.g., A, B, or E), it must visually resemble a manifestation of that letter. Here, then, the critical manipulation involves the similarity of the *substituted* digit to the original letter, and form does matter (e.g., 4 resembles A, but 5 does not).

The fact that masked priming is insensitive to the similarity of the form of the allograph letters in the prime and target follows naturally from the idea that the prime letters contribute evidence for the identity of the word, and the letters in the target carry on contributing further evidence. An uppercase A and a lower case a provide equally good evidence for any word containing the abstract letter identity representing the first letter of the alphabet. This idea is formalized in Norris and Kinoshita’s (2008) account of masked priming, in which masked-priming effects are explained in terms of the prime and the target both contributing evidence to the decision required by the task. Norris and Kinoshita (2008, Exps. 2 and 3) studied letter similarity effects using the same–different task. In this task, participants are instructed to decide whether the target is the same as or different from the referent that is presented in advance for 1 s. When the referent was always in the opposite case from the target (the “cross-case” same–different task) and participants were instructed to respond “Same” irrespective of the difference in case (e.g., responding “Same” to the target A and the referent a), the visual similarity of the masked prime and the target (e.g., a and A are dissimilar; c and C are similar) did not modulate the size of priming. That is, priming appeared to be driven by abstract letter identity, and not by the physical form of the letters. The same prime–target pairs showed a completely different pattern of priming when the task was changed. When participants were instructed to respond “Same” only if the referent and the target were the same letter in the same case (e.g., responding “Same” to the target A and the referent A, and responding “Different” to the target A and the referent a), priming was abolished for the visually dissimilar prime–target letter pairs. These results led Norris and Kinoshita to argue that priming was not an automatic consequence of the visual similarity between the prime and target, but is guided by the demands of the task.

So, in the tasks of lexical decision or same–different comparisons of letter identity (letter name), priming is determined by abstract letter identity. The letters a and c in the prime contribute the same amount of evidence toward the abstract letter identities A/a and C/c, respectively, irrespective of the similarity of a form to the letter in the target (i.e., a is dissimilar in form to A; c is similar in form to C). However, in the case of leet priming, there is competition between the different possible interpretations of the letters in the prime for which digits are substituted (the similarity of the digits to the letter—i.e., the similarity of 4 to A, as compared with 5 to A). This changes the evidence that is being contributed to the abstract representation of the letter a/A. The digit 5, being less similar to A than is 4, will contribute less evidence for the abstract letter a/A. The similarity between the digit and the letter therefore modulates priming. In the experiment to be reported here, we focused on trying to understand exactly how this competition process operates. Specifically, we compared leet priming with an analogous manipulation of similarity in which similar or dissimilar *letters* were substituted for the letters. Would a visually similar letter (e.g., H substituting for A) produce more priming than a visually dissimilar letter (e.g., D substituting for A)?

Before turning to this experiment, we should note some methodological differences between the allograph experiments and the leet-priming experiments that may have contributed to the disparate results. One is the length, and hence the neighborhood density, of the target words used. The allograph studies (e.g., Bowers et al., 1998) used short words (which are necessarily in dense neighborhoods), whereas the leet-priming studies (e.g., Perea et al., 2008) used six- to eight-letter (Spanish) words. Given that there is evidence that orthographic priming effects in lexical decision are small and weak for short words in dense lexical neighborhoods (the “target density constraint”; Forster, Davis, Schoknecht, & Carter, 1987), it may be that the allograph studies using shorter words were not sensitive enough to pick up an effect of visual similarity on masked priming. Second, and potentially more important, in the leet-priming study by Perea et al. (2008), both the prime and target were presented in uppercase letters (though in different-size fonts: 10-point Courier for the prime and 12-point Courier for the target, so that the physical overlap was not complete), so it is arguable that the “visually similar” leet prime and the target were overall *physically* more similar than in the allograph studies, in which the prime and target were presented in different cases (consider, e.g., M4T3R14L–MATERIAL vs. material–MATERIAL). It should be noted that some data argue against this possibility. Kinoshita and Norris (2011, Exp. 1) observed that the size of the identity-priming effects in lexical decision for word targets presented in mIxEd CaSe (e.g., wEaPoN, presented in Courier New 12-point font) did not differ when the prime presented in lowercase letters was in the same font as the target (e.g., weapon, in 12-point Courier New) or in a different font (e.g., weapon in Arial 10-point font). That is, the greater physical similarity between the letters in the prime and the target did not impact the size of priming, and by extension, Perea et al.’s (2008) leet-priming results are also unlikely to be due to low-level physical similarity. Nonetheless, it is possible that some or all of these methodological differences in combination may explain why visual similarity modulated priming in the leet-priming studies but not in the allograph studies.

The aim of the present study was to investigate the effects of visual similarity between letters on priming. Would substituting visually similar letters for the letters in a prime (e.g., A with H, or B with R) produce more priming than substituting visually dissimilar letters for them (e.g., A with D, or B with W), as in the leet-priming effect? To test this, we used four critical prime conditions resulting from a factorial combination of visual similarity (similar vs. dissimilar) and substitution type (digit vs. letter). In the visually similar digit prime, the critical letters were replaced with visually similar digits (e.g., A/4, B/8, to produce 484NDON–abandon), and in the visually dissimilar digit condition, the critical letters were replaced with visually dissimilar digits (e.g., 676NDON–abandon). On the basis of the leet-priming results reported previously, we expected the visually similar digit primes to produce greater priming than the dissimilar digit primes. The key comparison involved the two letter substitution prime conditions. In the visually similar letter prime, the critical letters were replaced with visually similar letters (e.g., A/H, B/R, for HRHNDON–abandon), and in the visually dissimilar letter prime, the critical letters were replaced by visually dissimilar letters (e.g., DWDNDON–abandon).

In the experiment, we also wished to eliminate the potential confounds between the allograph studies and the leet-priming studies described above. To this end, the visual similarity manipulation was applied to the same base letters; that is, the same letters in the prime were replaced with both visually similar/dissimilar digits (leet prime) and letters. Also, we used seven-letter-long target words (which have few neighbors), so as to maximize the opportunity for observing orthographic priming effects. To minimize physical overlap in the stimuli, we presented the prime and target in different cases.

In addition, to rule out the possibility that any difference between the digits and letters might be attributed to a stronger manipulation of visual similarity for one item type (e.g., 4 may be more visually similar to A than H is to A), we took measures to equate the similarity of the letter and digit substitutions to the base letter. We first consulted a number of letter confusion matrices (reviewed in Mueller & Weidemann, 2012) to select letters that were very confusable (for the similar-letter condition) and were least confusable (for the dissimilar-letter condition) with the base letter. We then presented these letters and digits as distractors and the base letter as the target in a two-alternative forced choice (2AFC) identification task. We used the identification data to select replacement letters and replacement digits, so that they were matched on confusability with the target letters. (The data from this identification task are presented in Appendix A.) We repeated the 2AFC identification task with the participants in our critical masked-priming experiment, to ensure that they also found the substitution digits and letters to be equally confusable with the target letter when all characters were presented singly.

## Method

### Participants

A group of 37 students from Macquarie University participated in the experiment in return for course credit.

### Design

For the experiment, we used the lexical decision task and manipulated the factor Prime Type (identity, letter similar, letter dissimilar, digit similar, digit dissimilar, and all letters different) within subjects. The dependent variables were response latency and error rate.

## Materials

The critical stimuli were 120 seven-letter words, containing at least three occurrences of the letters A, I, S, or B. These letters were chosen because they had been used in previous leet-priming studies (e.g., Kinoshita & Lagoutaris, 2010; Perea et al., 2008; Perea, Duñabeitia, Pollatsek, & Carreiras, 2009) and had digits that resembled them (A/4, I/1, S/5, and B/8). In addition to the visually similar digits, for each of the letters, we chose a visually dissimilar digit (A/6, I/2, S/7, and B/7), a visually similar letter (A/H, I/L, S/E, and B/R), and a visually dissimilar letter (A/D, I/G, S/D, and B/W). These letters were chosen from a wider range of items selected by consulting Mueller and Weidemann’s (2012) letter confusion matrices, which were then tested in a 2AFC identification task conducted to equate the digits and letters on their degrees of similarity to the base letter. The procedure of the 2AFC perceptual identification task was identical to that conducted in the main experiment described below, except that the targets were presented for 53 ms. The data for the pilot 2AFC perceptual identification task are presented in Appendix A.

The 120 critical words were selected from the English Lexicon Project (ELP) database (Balota et al. 2007, available at http://elexicon.wustl.edu/) and had a maximum of two orthographic neighbors (range 0–2, mean 0.4), as defined by the *N* metric (Coltheart, Davelaar, Jonasson, & Besner, 1977). This was done to maximize the opportunity for observing orthographic priming effects, since short words from dense lexical neighborhoods show small priming effects in the lexical decision task. We chose words with a minimum mean lexical decision accuracy of .88 (mean = .97). This was done to ensure that the stimuli were known to the participants as words. The words ranged in frequency (15–206, mean = 49.1 per million, based on Kučera & Francis, 1967; 0.29–194.8, mean = 22.5 per million, based on Subtlex frequency—Brysbaert & New, 2009; 6.57–12.42, mean = 9.4 log HAL frequency). Examples are *abandon* and *optimal*.

For each word, six primes were generated. The critical prime conditions were a factorial combination of the factors Similarity (similar vs. dissimilar) and Substitution Type (letter vs. digit). Each target word contained the letters A, I, S, or B. Within each word, each occurrence of these four letters was then replaced with one of the four substitution characters. In the *similar-letter* prime, the letters were replaced with letters that were visually similar to the target letters: H for A, L for I, E for S, and R for B—for example, HRHNDON for ABANDON. In the *dissimilar-letter* prime, the letters were replaced with letters that were visually dissimilar to the target letters: D for A, G for I, D for S, and W for B—for example, DWDNDON for ABANDON. In the *similar-digit* prime, the letters were replaced with digits that were visually similar to the target letters: 4 for A, 1 for I, 5 for S, and 8 for B—for example, 484NDON for ABANDON. In the *dissimilar-digit* prime, the letters were replaced with digits that were visually dissimilar to the target letters: 6 for A, 2 for I, 7 for S, and 7 for B—for example, 676NDON for ABANDON. The remaining two primes were an *identity* prime—for example, ABANDON for ABANDON—and an *all-letter-different (ALD)* prime, which was a different word that had no letters in common with the target—for example, PRODUCT for ABANDON. All of the primes were presented in uppercase letters, and the targets were presented in lowercase letters. The target words and primes are listed in Appendix B.

The 120 target words were divided into six sets of 20, matched on mean frequency. Six list versions were constructed for the purpose of counterbalancing the assignments of sets to the six prime types using a Latin square, so that within a list, each target word occurred only once, and across the six lists, each word appeared in each of the six prime conditions once.

In addition to the critical target words, 120 seven-letter nonwords were selected from the ELP database. They were all orthographically legal and matched item-by-item to the words for *N*—for example, ABEMISK, FENGILE. The same six types of primes used with the word targets (identity, similar letter, dissimilar letter, similar digit, dissimilar digit, and all letters different) were generated for the nonword targets, according to the same procedure. Six list versions were constructed for the purpose of counterbalancing the assignments of sets to the six prime types using a Latin square, so that within a list, each target nonword occurred only once, and across the six lists, each nonword appeared in each of the six prime conditions once.

In addition, 12 practice and initial buffer items were presented, which were selected according to the same criteria as the test stimuli. These items were not included in the analysis.

### Apparatus and procedure

Participants were tested in groups of one to four, seated approximately 60 cm in front of a CRT monitor, upon which the stimuli were presented. Each participant completed 240 test trials consisting of 120 word and 120 nonword trials, presented in two half-blocks (with each half-block containing equal numbers of word and nonword trials and equal numbers of items from the different prime conditions), with a self-paced break between the blocks. A different random order of trials was generated for each participant.

#### Lexical decision task

Participants were instructed at the outset of the experiment that on each trial they would be presented with a letter string in lowercase letters following a warning signal consisting of # signs, and that their task was to decide whether the letter string was a word or a nonword as quickly and accurately as possible. No mention was made of the presence of primes. Participants were instructed to press a key on a response pad marked “+” for “Word” and a key marked “-” for “Nonword” responses.

The stimulus presentation and data collection were achieved through the use of the DMDX display system, developed by K. I. Forster and J. C. Forster at the University of Arizona (Forster & Forster, 2003). Stimulus display was synchronized to the screen refresh rate (13.3 ms).

Each trial started with the presentation of a forward mask, consisting of seven # signs, for 500 ms in the center of the screen. It was replaced by the prime in uppercase letters, presented for 40 ms, then by the target, presented in lowercase letters for a maximum of 2,000 ms, or until the participant’s response. (Note that it was necessary to present the prime in uppercase letters, because the letters that the digits resembled were all uppercase letters—e.g., 4–A, 8–B, etc.) The forward mask and primes were presented in Courier New 10-point font, and the target was in Courier New 12-point font, so that the target effectively backward-masked the prime. Participants were given feedback (the message “Wrong response” presented on the screen) only when they made an error.

#### 2AFC identification task

Immediately after the lexical decision task, participants performed an identification task. The purpose of this task was to verify the perceived similarity of the substituted letter/digit to the target letter. On each trial, following a forward mask consisting of three # signs presented for 500 ms, a letter or a digit was presented flanked by % signs (e.g., “%A%”) for 40 ms, and followed immediately by a backward mask consisting of three # and @ signs overlaid on each other. Two alternatives, one a target (e.g., A) and the other a distractor (e.g., 4), were presented simultaneously to the right and left of the backward mask, and remained on the screen until the participant’s response. Participants were instructed that they had 10 s to indicate which of the two alternatives had been presented as the target, by pressing a key corresponding to its location (left or right). No feedback was given. A total of 192 trials were presented, with each trial containing one of the critical letters (A, I, S, or B) and its substituted counterpart (similar letter, dissimilar letter, similar digit, or dissimilar digit). The critical letter and its substituted counterpart were presented equally often as the target to be identified, and the correct alternative was presented on the right or the left equally often. A full counterbalancing therefore required 64 trials (4 pairings of a critical letter and substituted counterpart × 4 critical letters × target/distractor presented × position of target/distractor), and each of these 64 trials was presented three times. A different random order of trials was generated for each participant.

## Results

### Lexical decision task

We analyzed the response times (RTs) from correct trials and the error rates for word targets using a linear mixed-effects model (Baayen, 2008). We did not analyze the nonword data, since they are insensitive to masked priming in the lexical decision task (see Norris & Kinoshita, 2008, for an explanation). The preliminary treatment of the correct RT data for this analysis was as follows. First, we examined the shape of the RT distribution (a total of 4,187 data points) and applied an inverse transformation (1/RT) to approximate a normal distribution, in order to meet the distributional assumption of the linear mixed-effects model. (We used the inverse transformation rather than the log transformation because the inverse transformation approximated the normal distribution better.) We excluded trials with RTs shorter than 300 ms (six data points). This cutoff for outliers was determined by inspecting the Q–Q plots of the inverse-transformed RTs. The dependent variable used in our analysis was “invRT,” defined as −1,000/RT: We multiplied 1/RT by −1,000 to maintain the direction of the effects (so that a larger invRT meant a slower response), and to avoid too many decimal places. We used the lme4 (Version 0.999999-2; Bates, Maelchler, & Bolker, 2013) package, as implemented in R Version 3.0.0 (R Development Core Team, 2013), to carry out the linear mixed-effect model analysis, treating Subjects and Items as crossed random factors. The *p* values reported here were estimated using the Markov-chain Monte Carlo sampling method (with the default 10,000 iterations), implemented in the languageR package (Version 1.4; Baayen, 2011).

Table 1 shows the mean RTs in the six prime conditions for the word targets as well as the nonword targets.

**Table 1:** Mean decision latencies (RTs, in milliseconds) and percent error rates (%E) in the experiment (lexical decision task)

Prime type	Example	RT	%E
Word target (abandon)			
Identity	ABANDON	504	4.1
Similar letter	HRHNDON	519	6.4
Dissimilar letter	DWDNDON	527	5.7
Similar digit	484NDON	509	4.7
Dissimilar digit	676NDON	530	6.9
ALD	PRODUCT	557	5.7
Nonword target (faxisum)			
Identity	FAXISUM	594	5.0
Similar letter	FHXLEUM	594	5.0
Dissimilar letter	FDXGDUM	597	4.6
Similar digit	F4X15UM	593	6.2
Dissimilar digit	F6X27UM	606	6.4
ALD	ICQUIDE	603	6.2

The primes were presented in uppercase letters in Courier New 10-point font, and the targets were presented in lowercase letters in Courier New 12-point font, so that the target effectively backward-masked the prime.

We first tested a statistical model that included, as fixed factors, Prime Type (referenced to the ALD prime), Log HAL Frequency, and prevRT (RT on previous trial), with the Subjects Intercept (37) and Target Intercept (120) as crossed random factors: invRT ~ Prime type + Log HAL frequency + prevRT + (1|subject) + (1|target).^2^ To examine the effect of the previous-trial RT, trials on which an error had been made on the previous trial were excluded from the analysis, resulting in 3,974 data observations. The model shows that the effect of log HAL frequency was significant, *t* = −6.875, *p* < .0001, as was prevRT, *t* = 12.975, *p* < .0001. Referenced to the ALD prime condition, all prime conditions were significantly faster: identity *t* = −10.494, *p* < .0001; similar letter *t* = −6.497, *p* < .001; dissimilar letter *t* = −4.968, *p* < .001; similar digit *t* = −9.743, *p* < .001; and dissimilar digit *t* = −5.014, *p* < .001. Referenced to the identity-prime condition, the 5-ms difference from the similar-digit prime condition was not significant, *t* = 0.794, *p* = .418, but the 15-ms difference from the similar-letter prime condition was, *t* = 3.953, *p* < .0001.

We then analyzed the critical four experimental conditions in a 2 × 2 factorial design with Similarity (similar vs. dissimilar) and Substitution Type (letter vs. digit) as fixed factors. The similarity effect was significant, *t* = −4.396, *p* < .0001, with similar primes facilitating responses to the targets. The substitution type effect was also significant, *t* = 2.216, *p* < .026, with the digit primes producing faster responses to the targets. Importantly, the interaction was significant, *t* = 2.131, *p* < .034, indicating that the similarity effect was greater for the digit primes than for the letter primes. Individual comparisons indicated that the 21-ms similarity effect for the digit primes was significant, *t* = −4.448, *p* < .001, but the 8-ms effect for the letter primes was not, *t* = −1.655, *p* = .0982.

Error rates were analyzed using the logit linear mixed-effects model (Jaeger, 2008) with Prime Type and Log HAL Frequency as fixed factors and the Subject Intercept and Word Intercept as crossed random factors. Log HAL frequency significantly reduced the error rate, *z* = −6.061, *p* < .0001. Referenced to the ALD prime, none of the prime conditions differed significantly from this condition. The analysis of the critical four prime conditions showed that neither the effect of similarity (*z* = −1.023, *p* = .306) nor substitution type (*z* = 0.229, *p* = .818), nor the interaction between the two (*z* = 1.614, *p* = .106), was significant.

### 2AFC identification task

Averaged across the four target letters (A, I, S, B), the mean error rates were 44.15 % for the similar-letter distractor, 41.17 % for the dissimilar-letter distractor, 43.52 % for the similar-digit distractor, and 41.38 % for the dissimilar digit distractor. We analyzed the error rates using the logit linear mixed-effects model (Jaeger, 2008), with Distractor Type (letter vs. digit) and Visual Similarity (similar vs. dissimilar) as fixed factors and Subject Intercept (37) and Target Intercept (4) as crossed random factors. Distractor type was nonsignificant, *z* = 0.168, *p* = .866, but similarity was significant, *z* = 2.214, *p* < .027. We found no interaction between the two, *z* = 0.364, *p* = .716. Thus, the identification data confirmed that the letter and digit distractors were equated on their perceived similarity to the target letter, with the similar distractors being perceived to be more similar to the target than the dissimilar distractors.

## Discussion

The results were clear cut, indicating a dissociation between the effects of visual similarity for digit primes and letter primes. Replicating previous leet-priming studies, the primes containing visually similar digits (e.g., 484NDON for ABANDON) produced robust priming, which was greater than that produced by the primes containing visually dissimilar digits (e.g., 676NDON for ABANDON). The priming effect produced by visually similar digit primes was equal in size to the identity-priming effect (ABANDON–abandon). It should be noted that, unlike the previous leet-priming studies that had presented the prime and target in the same case (both uppercase), the targets were presented in lowercase letters; hence, the leet-priming effect observed here cannot be attributed to a physical overlap between the prime and target. In contrast to the digit primes, the letter primes showed little effect of visual similarity: Similar-letter primes (e.g., HRHNDON) produced no more priming than did dissimilar-letter primes (e.g., DWDNDON). The comparison to the identity prime condition further confirmed that the visually similar letter primes produced significantly less priming than did the identity primes. The 2AFC identification task indicated that the dissociative effect of visual similarity was not due to the similar-digit distractor being perceptually more similar than the similar-letter distractor to the base letter: The letter and digit distractors (e.g., H and 4, respectively) used in the masked prime were perceived as being equally confusable with the base letter (e.g., A).

As far as we are aware, this was the first study to examine the effects of visual similarity on masked priming with substituted-letter primes.^3^ Unlike the digit primes, which showed greater priming for visually similar items (replicating the previous findings of leet priming), visual similarity had little impact on the priming produced by the substituted-letter primes. How can this dissociation be explained, and what are the implications for the current models of visual word recognition?

One potential solution to explaining this dissociation would be to propose that a top-down feedback mechanism, available selectively for letter stimuli, normalizes the shape of the ambiguous form. Indeed, such a view was proposed by Perea, Duñabeitia, Pollatsek, and Carreiras (2009) to explain their findings using the same–different match task. In their study, primes containing leet digits (e.g., V35Z3D) facilitated matches for letter-string targets (e.g., VESZED), but primes containing leet letters (e.g., 9ES7E2) did not facilitate matches for digit-string targets (e.g., 935732). This top-down feedback account was, however, ruled out by Kinoshita and Lagoutaris (2010), who showed that the asymmetry in leet priming was due to the difference in the ease of maintaining in visual working memory a long reference sequence of random digits (e.g., 935732) versus a well-formed pseudoword (e.g., VESZED). In order to perform a same–different match, the target must be compared against a representation of the reference string maintained in visual working memory, the capacity of which is generally assumed to be four objects (Vogel, Woodman, & Luck, 2001). Kinoshita and Lagoutaris thus reasoned that it would be difficult to maintain an accurate representation of a random sequence of six digits, but it may be possible to maintain a sequence of six letters that form a pseudoword by means of chunking. Consistent with this theory, the size of the *identity*-priming effect was also reduced for the six-digit strings relative to the six-letter pseudowords. Critically, Kinoshita and Lagoutaris showed that the asymmetry in leet priming was eliminated when (1) the reference string was a short sequence of four digits (e.g., 2157) or a four-letter pseudoword (e.g., MISF), and (2) the reference string was a long sequence of six random digits (e.g., 214637) or a six-letter sequence of random letters (e.g., OIAUEQ). These findings provide no support for the view that a top-down feedback mechanism is available selectively for letter stimuli.

To explain the present finding of the dissociative effect of visual similarity, we suggest that the key difference between the digit and letter primes is the presence of a (letter) competitor. In the case of the visually similar digit primes (e.g., 4), the target letter (e.g., A) is the best-matching letter. This is not the case for the letter primes: The letter itself (e.g., H) is the best-matching letter, better than the target letter (e.g., A) that it substitutes for. Thus, whereas a visually similar *digit* (4) may be taken as an instance of the target letter it is substituted for (A), a visually similar *letter* (H) cannot be—the latter would be just as poor an instance of the target letter as would a letter that is visually dissimilar to the target (e.g., D). Consequently, a visually similar digit prime (e.g., 484NDON) can facilitate the retrieval of a lexical representation corresponding to the target word (i.e., produce priming) better than a visually dissimilar digit prime, but a prime containing letter substitutions would produce similar amounts of priming, irrespective of the visual similarity of the substituted letters to the target letters.

This notion of competition that exists between letters may be implemented within an interactive-activation framework (adopted by almost all models of visual word recognition, including DRC (Coltheart et al., 2001), MROM (Grainger & Jacobs, 1996), the spatial-coding model (Davis, 2010), and the various open-bigram models)^4^ or a Bayesian framework (the Bayesian Reader model (Norris, 2006), and its successors, the noisy slot model (Norris et al., 2010) and the noisy channel model (Norris & Kinoshita, 2012). In the interactive-activation framework, competition is typically implemented in the form of reciprocal inhibitory connections: For example, the letter representations A and H may be connected by a bidirectional inhibitory link, such that the activation of one drives down the activation of the other. Such within-level mutual inhibition is common in the interactive-activation framework and reflects the assumption that a representation cannot be two different things simultaneously—it is either the letter A or H; it cannot simultaneously be both A *and* H. Competition between perceptually similar items is also an intrinsic feature within a Bayesian framework. In order to recognize a letter, the reader must accumulate enough evidence to distinguish that letter from a perceptually similar letter. According to Bayes’s theorem, the probability of each letter is a function of the evidence for that letter (called the “likelihood”), divided by the evidence for all of the other letters. Obviously, the greater the evidence for an alternative letter, the more competition there would be.

A question raised by this analysis is why the priming data showed that effectively, no competition took place between the letter A and the digit 4. Just as a representation cannot simultaneously be A and H, it cannot be both A and 4, yet the priming data showed that 4 was taken as an instance of A, but that H was not. This cannot be attributed to the greater physical resemblance between 4 and A than between H and A, because the 2AFC perceptual identification data showed that the visually similar digits were just as difficult to discriminate from the base letter as the visually similar letters.

We suggest that the difference between digits and letter primes observed in the present lexical decision experiment follows naturally from a Bayesian framework, which assumes that what serves as an effective competitor is guided by the task. In the lexical decision task, the goal of the task is to decide whether the letter string is a word, and here, the targets were all strings of letters. This involves accumulating the evidence for the hypothesis “which letter,” not “which letter or digit?” The digit 4 does not serve as a competitor to the letter A because it is written out of contention by the nature of the task (and the targets used): In the lexical decision task, the task-guided expectation is that the input contains only letters, not digits. The visually similar digit 4 contributes almost as much evidence as the letter A for the hypothesis that it is the letter A, because the hypothesis does not consider the possibility that it is a digit. The way that priming is modulated by the nature of the decision rather than simply by the similarity of the form of the stimulus is analogous to the pattern of letter priming reported by Norris and Kinoshita (2008), discussed in the introduction. Using the same–different task, they found that the similarity of the prime and the target letter only played a role when the task was to judge whether the referent and the target were physically identical. In that case, the decision about the form of the stimulus must necessarily be influenced by the physical overlap between prime and target. When the task was to judge whether the letters had the same name, the exact form of the stimulus was no longer relevant, and no effect of the similarity of the prime and the target letters emerged. This account also explains why there was no asymmetry in leet priming for digit primes versus letter primes in the same–different task (Kinoshita & Lagoutaris, 2010), since in that task both digit and letter reference strings were used.

In closing, we note a parallel in the emerging consensus regarding the role of the so-called “visual word form area” (VWFA). Brain imaging studies have shown that an area in the left fusiform gyrus, along the ventral pathway, supports the perception of abstract orthographic forms (see, e.g., Dehaene & Cohen, 2011; Dehaene et al., 2004), and lesions at or near this region have long been known to result in a relatively specific impairment of fluent reading (“pure alexia”; Dejerine, 1892). However, spirited debate has attended whether this area is selectively or preferentially responsive to letters rather than to other visual objects (e.g., Price & Devlin, 2003; Vogel, Petersen, & Schlaggar, 2012). In this context, it is relevant to note that there is growing recognition that the finding of a “wordlikeness gradient” (i.e., greater hemodynamic activity for more wordlike stimulus) in the VWFA is task-dependent (e.g., Wang, Yang, Shu, & Zevin, 2011) and that the role of this area for reading is not static. Dehaene and Cohen noted that “purely bottom-up visual factors are not the sole determinant of its organization” (p. 260), and A. C. Vogel et al. wrote that “we are arguing that the (putative) VWFA may not be best conceived of as a ‘letter’ or ‘word’ area. Instead, we hypothesize that left OT cortex becomes useful for processing words and letters due to its information processing properties” (p. 2730). Our account of the dynamic nature of the visual similarity of letters and digits is entirely consistent with these views.

## Appendixes

### Appendix A

**Table 2:** Stimuli used and the error rates (ER) in the two-alternative forced choice (2AFC) perceptual identification task used to select the letters used as substitutes in the prime

Target letter	Similar		Dissimilar	
	Distractor	ER	Distractor	ER
A				
Digit distractor	4	42.9	6	22.2
Letter distractors	H	31.7	D	24.6
	R	31.7	C	30.9
E				
Digit distractor	3	32.5	9	31.7
Letter distractors	F	50	Q	28.6
	B	39.7	C	24.6
I				
Digit distractor	1	30.2	2	18.3
Letter distractors	L	26.2	G	14.3
	T	15.9	R	21.4
S				
Digit distractor	5	35.7	7	31.7
Letter distractors	E	31.7	D	28.6
	Z	23.8	I	55.6
B				
Digit distractor	8	39.7	7	29.4
Letter distractor	R	34.9	W	26.9
	P	36.5	V	33.3

In this 2AFC identification task, 21 participants, in addition to the participants in the experiment described in the article, were tested. The procedure was identical to that described in the Method section of the main experiment, except that the prime duration was 53 ms, instead of 40 ms. On the basis of this data, the letter distractors in the first row (e.g., for the target A, the letters H and D) were selected as the letter substitutions to be used in the lexical decision experiment. The letter E was not used in the main experiment.

### Appendix B

**Table 3:** List of the critical stimuli used in the lexical decision experiment

Target	Identity	Digitsim	Digitdis	Letsim	Letdis	ALD
abandon	ABANDON	484NDON	676NDON	HRHNDON	DWDNDON	PRODUCT
absence	ABSENCE	485ENCE	677ENCE	HREENCE	DWDENCE	VARIETY
address	ADDRESS	4DDRE55	6DDRE77	HDDREEE	DDDREDD	MORNING
anxious	ANXIOUS	4NX1OU5	6NX2OU7	HNXLOUE	DNXGOUD	TRACTOR
balance	BALANCE	84L4NCE	76L6NCE	RHLHNCE	WDLDNCE	POTTERY
clarity	CLARITY	CL4R1TY	CL6R2TY	CLHRLTY	CLDRGTY	WESTERN
fashion	FASHION	F45H1ON	F67H2ON	FHEHLON	FDDHGON	CENTURY
husband	HUSBAND	HU584ND	HU776ND	HUERHND	HUDWDND	REFUSAL
initial	INITIAL	1N1T14L	2N2T26L	LNLTLHL	GNGTGDL	STRANGE
justify	JUSTIFY	JU5T1FY	JU7T2FY	JUETLFY	JUDTGFY	PIONEER
massive	MASSIVE	M4551VE	M6772VE	MHEELVE	MDDDGVE	SERVANT
minimal	MINIMAL	M1N1M4L	M2N2M6L	MLNLMHL	MGNGMDL	CLIMATE
pacific	PACIFIC	P4C1F1C	P6C2F2C	PHCLFLC	PDCGFGC	JOURNEY
plastic	PLASTIC	PL45T1C	PL67T2C	PLHETLC	PLDDTGC	SOMEHOW
success	SUCCESS	5UCCE55	7UCCE77	EUCCEEE	DUCCEDD	MOVABLE
traffic	TRAFFIC	TR4FF1C	TR6FF2C	TRHFFLC	TRDFFGC	OPTIMUM
usually	USUALLY	U5U4LLY	U7U6LLY	UEUHLLY	UDUDLLY	CONDUCT
utopian	UTOPIAN	UTOP14N	UTOP26N	UTOPLHN	UTOPGDN	MYSTERY
visible	VISIBLE	V1518LE	V2727LE	VLELRLE	VGDGWLE	NERVOUS
worship	WORSHIP	WOR5H1P	WOR7H2P	WOREHLP	WORDHGP	BUILDER
ability	ABILITY	481L1TY	672L2TY	HRLLLTY	DWGLGTY	VOLTAGE
caution	CAUTION	C4UT1ON	C6UT2ON	CHUTLON	CDUTGON	PROCEED
cavalry	CAVALRY	C4V4LRY	C6V6LRY	CHVHLRY	CDVDLRY	ACCOUNT
curious	CURIOUS	CUR1OU5	CUR2OU7	CURLOUE	CURGOUD	MEANING
dynamic	DYNAMIC	DYN4M1C	DYN6M2C	DYNHMLC	DYNDMGC	HELPFUL
embassy	EMBASSY	EM8455Y	EM7677Y	EMRHEEY	EMWDDDY	DISPUTE
insight	INSIGHT	1N51GHT	2N72GHT	LNELGHT	GNDGGHT	TEXTURE
instant	INSTANT	1N5T4NT	2N7T6NT	LNETHNT	GNDTDNT	FORMULA
minimum	MINIMUM	M1N1MUM	M2N2MUM	MLNLMUM	MGNGMUM	TEXTILE
mission	MISSION	M1551ON	M2772ON	MLEELON	MGDDGON	COTTAGE
mustard	MUSTARD	MU5T4RD	MU7T6RD	MUETHRD	MUDTDRD	WELCOME
obvious	OBVIOUS	O8V1OU5	O7V2OU7	ORVLOUE	OWVGOUD	DAYTIME
optimal	OPTIMAL	OPT1M4L	OPT2M6L	OPTLMHL	OPTGMDL	RESPECT
painful	PAINFUL	P41NFUL	P62NFUL	PHLNFUL	PDGNFUL	BROTHER
physics	PHYSICS	PHY51C5	PHY72C7	PHYELCE	PHYDGCD	NETWORK
quality	QUALITY	QU4L1TY	QU6L2TY	QUHLLTY	QUDLGTY	LITERAL
serious	SERIOUS	5ER1OU5	7ER2OU7	EERLOUE	DERGOUD	HIGHEST
similar	SIMILAR	51M1L4R	72M2L6R	ELMLLHR	DGMGLDR	REPLACE
station	STATION	5T4T1ON	7T6T2ON	ETHTLON	DTDTGON	INVOLVE
tourist	TOURIST	TOUR15T	TOUR27T	TOURLET	TOURGDT	DESTROY
arrival	ARRIVAL	4RR1V4L	6RR2V6L	HRRLVHL	DRRGVDL	DESPITE
circuit	CIRCUIT	C1RCU1T	C2RCU2T	CLRCULT	CGRCUGT	QUARREL
consist	CONSIST	CON515T	CON727T	CONELET	CONDGDT	STRETCH
crucial	CRUCIAL	CRUC14L	CRUC26L	CRUCLHL	CRUCGDL	SUBJECT
darling	DARLING	D4RL1NG	D6RL2NG	DHRLLNG	DDRLGNG	SENATOR
dignity	DIGNITY	D1GN1TY	D2GN2TY	DLGNLTY	DGGNGTY	PAYMENT
discuss	DISCUSS	D15CU55	D27CU77	DLECUEE	DGDCUDD	COMPLEX
disease	DISEASE	D15E45E	D27E67E	DLEEHEE	DGDEDDE	PROTEIN
drawing	DRAWING	DR4W1NG	DR6W2NG	DRHWLNG	DRDWGNG	INTENSE
halfway	HALFWAY	H4LFW4Y	H6LFW6Y	HHLFWHY	HDLFWDY	JUSTICE
highway	HIGHWAY	H1GHW4Y	H2GHW6Y	HLGHWHY	HGGHWDY	RELEASE
instead	INSTEAD	1N5TE4D	2N7TE6D	LNETEHD	GNDTEDD	CEILING
maximum	MAXIMUM	M4X1MUM	M6X2MUM	MHXLMUM	MDXGMUM	PICTURE
passion	PASSION	P4551ON	P6772ON	PHEELON	PDDDGON	DEFENSE
reality	REALITY	RE4L1TY	RE6L2TY	REHLLTY	REDLGTY	STUDENT
sponsor	SPONSOR	5PON5OR	7PON7OR	EPONEOR	DPONDOR	DENSITY
stadium	STADIUM	5T4D1UM	7T6D2UM	ETHDLUM	DTDDGUM	PITCHER
surplus	SURPLUS	5URPLU5	7URPLU7	EURPLUE	DURPLUD	MEETING
transit	TRANSIT	TR4N51T	TR6N72T	TRHNELT	TRDNDGT	PAYROLL
various	VARIOUS	V4R1OU5	V6R2OU7	VHRLOUE	VDRGOUD	NOWHERE
billion	BILLION	81LL1ON	72LL2ON	RLLLLON	WGLLGON	COURAGE
cabinet	CABINET	C481NET	C672NET	CHRLNET	CDWGNET	PRIVATE
contain	CONTAIN	CONT41N	CONT62N	CONTHLN	CONTDGN	FALLOUT
crystal	CRYSTAL	CRY5T4L	CRY7T6L	CRYETHL	CRYDTDL	INCLUDE
finance	FINANCE	F1N4NCE	F2N6NCE	FLNHNCE	FGNDNCE	NUCLEAR
imagine	IMAGINE	1M4G1NE	2M6G2NE	LMHGLNE	GMDGGNE	HERSELF
inquiry	INQUIRY	1NQU1RY	2NQU2RY	LNQULRY	GNQUGRY	DIVORCE
mankind	MANKIND	M4NK1ND	M6NK2ND	MHNKLND	MDNKGND	REVERSE
marshal	MARSHAL	M4R5H4L	M6R7H6L	MHREHHL	MDRDHDL	HUNDRED
million	MILLION	M1LL1ON	M2LL2ON	MLLLLON	MGLLGON	COMFORT
organic	ORGANIC	ORG4N1C	ORG6N2C	ORGHNLC	ORGDNGC	GENUINE
passage	PASSAGE	P4554GE	P6776GE	PHEEHGE	PDDDDGE	SHERIFF
session	SESSION	5E551ON	7E772ON	EEEELON	DEDDGON	TROUBLE
sizable	SIZABLE	51Z48LE	72Z67LE	ELZHRLE	DGZDWLE	TEACHER
stomach	STOMACH	5TOM4CH	7TOM6CH	ETOMHCH	DTOMDCH	VETERAN
summary	SUMMARY	5UMM4RY	7UMM6RY	EUMMHRY	DUMMDRY	CAREFUL
suspect	SUSPECT	5U5PECT	7U7PECT	EUEPECT	DUDPECT	FORTUNE
tobacco	TOBACCO	TO84CCO	TO76CCO	TORHCCO	TOWDCCO	FAILURE
unusual	UNUSUAL	UNU5U4L	UNU7U6L	UNUEUHL	UNUDUDL	MACHINE
useless	USELESS	U5ELE55	U7ELE77	UEELEEE	UDELEDD	DEVELOP
amazing	AMAZING	4M4Z1NG	6M6Z2NG	HMHZLNG	DMDZGNG	OUTDOOR
assault	ASSAULT	4554ULT	6776ULT	HEEHULT	DDDDULT	SOLDIER
classic	CLASSIC	CL4551C	CL6772C	CLHEELC	CLDDDGC	TANGENT
display	DISPLAY	D15PL4Y	D27PL6Y	DLEPLHY	DGDPLDY	REQUIRE
exhibit	EXHIBIT	EXH181T	EXH272T	EXHLRLT	EXHGWGT	OBSCURE
fiction	FICTION	F1CT1ON	F2CT2ON	FLCTLON	FGCTGON	COMPARE
finally	FINALLY	F1N4LLY	F2N6LLY	FLNHLLY	FGNDLLY	PLASTER
library	LIBRARY	L18R4RY	L27R6RY	LLRRHRY	LGWRDRY	SECULAR
musical	MUSICAL	MU51C4L	MU72C6L	MUELCHL	MUDGCDL	HARMONY
natural	NATURAL	N4TUR4L	N6TUR6L	NHTURHL	NDTURDL	SURFACE
optical	OPTICAL	OPT1C4L	OPT2C6L	OPTLCHL	OPTGCDL	HOSTILE
primary	PRIMARY	PR1M4RY	PR2M6RY	PRLMHRY	PRGMDRY	YOUNGER
pursuit	PURSUIT	PUR5U1T	PUR7U2T	PUREULT	PURDUGT	FACULTY
radical	RADICAL	R4D1C4L	R6D2C6L	RHDLCHL	RDDGCDL	IMPULSE
realism	REALISM	RE4L15M	RE6L27M	REHLLEM	REDLGDM	NOTABLE
satisfy	SATISFY	54T15FY	76T27FY	EHTLEFY	DDTGDFY	GESTURE
scholar	SCHOLAR	5CHOL4R	7CHOL6R	ECHOLHR	DCHOLDR	LARGEST
suicide	SUICIDE	5U1C1DE	7U2C2DE	EULCLDE	DUGCGDE	RESPOND
typical	TYPICAL	TYP1C4L	TYP2C6L	TYPLCHL	TYPGCDL	FORGIVE
warrant	WARRANT	W4RR4NT	W6RR6NT	WHRRHNT	WDRRDNT	THEOREM
airport	AIRPORT	41RPORT	62RPORT	HLRPORT	DGRPORT	EDITION
attract	ATTRACT	4TTR4CT	6TTR6CT	HTTRHCT	DTTRDCT	EMPEROR
bathing	BATHING	84TH1NG	76TH2NG	RHTHLNG	WDTHGNG	LECTURE
capable	CAPABLE	C4P48LE	C6P67LE	CHPHRLE	CDPDWLE	HORIZON
capital	CAPITAL	C4P1T4L	C6P2T6L	CHPLTHL	CDPGTDL	UNIFORM
despair	DESPAIR	DE5P41R	DE7P62R	DEEPHLR	DEDPDGR	FREIGHT
distant	DISTANT	D15T4NT	D27T6NT	DLETHNT	DGDTDNT	CONCERN
foolish	FOOLISH	FOOL15H	FOOL27H	FOOLLEH	FOOLGDH	SPEAKER
furnish	FURNISH	FURN15H	FURN27H	FURNLEH	FURNGDH	SCIENCE
gradual	GRADUAL	GR4DU4L	GR6DU6L	GRHDUHL	GRDDUDL	BATTERY
holiday	HOLIDAY	HOL1D4Y	HOL2D6Y	HOLLDHY	HOLGDDY	CHICKEN
liberal	LIBERAL	L18ER4L	L27ER6L	LLRERHL	LGWERDL	SUPPOSE
liberty	LIBERTY	L18ERTY	L27ERTY	LLRERTY	LGWERTY	COUNTER
logical	LOGICAL	LOG1C4L	LOG2C6L	LOGLCHL	LOGGCDL	FIFTEEN
message	MESSAGE	ME554GE	ME776GE	MEEEHGE	MEDDDGE	QUARTER
missile	MISSILE	M1551LE	M2772LE	MLEELLE	MGDDGLE	ETHICAL
mistake	MISTAKE	M15T4KE	M27T6KE	MLETHKE	MGDTDKE	ACADEMY
opinion	OPINION	OP1N1ON	OP2N2ON	OPLNLON	OPGNGON	COLONEL
qualify	QUALIFY	QU4L1FY	QU6L2FY	QUHLLFY	QUDLGFY	SLENDER
vicious	VICIOUS	V1C1OU5	V2C2OU7	VLCLOUE	VGCGOUD	CONCEPT

Target = target word, Identity = identity prime, Digitsim = similar-digit prime, Digitdis = dissimilar-digit prime, Letsim = similar-letter prime, Letdis = dissimilar-letter prime, ALD = all-letter-different control prime

## Footnotes

1 Bowers et al. (1998) further reported that in contrast to the word recognition tasks, in single-letter recognition tasks (e.g., the alphabet decision task), little masked priming was apparent for abstract letter identities. This conclusion seems to have been premature, because robust priming effects for abstract letter identities have been found in the same–different task, which is not subject to the stimulus–response mapping strategy in other letter recognition tasks that have been used (see Kinoshita & Kaplan, 2008, for details).

2 We also tested models containing the same fixed factors and subject and item random slopes on the Prime Type factor. Since the model with subject and item random slopes did not converge and the model with subject random slopes did not produce a better fit than the model with random intercepts [*χ* ^2^(20) = 25.389, *p* = .1869], we report the simpler model with random intercepts.

3 Recently, Perea and Panadero (2013) reported a similar absence of letter similarity effect with nonword *targets*. The authors found that adults and typically developing young readers were insensitive to the visual similarity of the substituted letter in a nonword target—e.g., *viotin* (in which the letter *t* is visually similar to the original letter *l*) was no more difficult to reject as a nonword than *viocin* (in which the letter *c* is visually dissimilar to *l*). Unlike in the present study, Perea and Panadero defined letter similarity in terms of whether the target and distractor letter shared an ascender/descender/neutral feature, however, and the confusability of the target and distractor letters was not verified empirically.

4 Although the present study was not specifically designed to distinguish between these models, the finding of a visual-similarity effect for the digit primes presents a problem for open-bigram models that claim that open bigram representations are specific to letter stimuli (see, e.g., Massol, Duñabeitia, Carreiras, & Grainger, 2013). Such models wrongly predict no difference between the similar- and dissimilar-digit primes (e.g., 484NDON and 676NDON for ABANDON), because they contain the same numbers of letters/open bigrams that overlap with the target.

## References

[CR1] Baayen RH (2008). Analyzing linguistic data: A practical introduction to statistics using R.

[CR2] Baayen, R. H. (2011). LanguageR: Data sets and functions with “Analyzing linguistic data: A practical introduction to statistics.” (Version 1.4). Retrieved from the CRAN archive: http://cran.r-project.org/src/contrib/Archive/languageR/

[CR3] Balota, D. A., Cortese, M. J., Hutchison, K. A., Neely, J. H., Nelson, D., Simpson, G. B., & Treiman, R. (2007). The English Lexicon Project: A web-based repository of descriptive and behavioral measures for 40,481 English words and nonwords. Retrieved from http://elexicon.wustl.edu/

[CR4] Bates, D., Maechler, M., & Bolker, B. (2013). lme4: Linear mixed-effects models using S4 classes [Software] (R package version 0.999999-2). Retrieved from http://cran.r-project.org/package=lme4

[CR5] Bowers JS, Vigliocco G, Haan R (1998). Orthographic, phonological, and articulatory contributions to masked letter and word priming. Journal of Experimental Psychology: Human Perception and Performance.

[CR6] Brysbaert M, New B (2009). Moving beyond Kučera and Francis: A critical evaluation of current word frequency norms and the introduction of a new and improved word frequency measure for American English. Behavior Research Methods.

[CR7] Carreiras M, Dunabeitia JA, Perea M (2007). READING WORDS, NUMB3R5 and $YMßOLS. Trends in Cognitive Sciences.

[CR8] Coltheart M, Davelaar E, Jonasson JT, Besner D, Dornic S (1977). Access to the internal lexicon. Attention and performance VI.

[CR9] Coltheart M, Rastle K, Perry C, Langdon R, Ziegler J (2001). DRC: A dual route cascaded model of visual word recognition and reading aloud. Psychological Review.

[CR10] Davis CJ (2010). The spatial coding model of visual word identification. Psychological Review.

[CR11] Dehaene S, Cohen L (2011). The unique role of the visual word form area in reading. Trends in Cognitive Sciences.

[CR12] Dehaene S, Jobert A, Naccache L, Ciuciu P, Poline JB, Le Bihan D, Cohen L (2004). Letter binding and invariant recognition of masked words: Behavioral and neuroimaging evidence. Psychological Science.

[CR13] Dejerine J (1892). Contribution à l’étude anatomo-pathologique et clinique des différentes variétés de cécité verbale. Comptes Rendus des Séances et Mémoires de la Société de Biologie.

[CR14] Forster KI, Davis C (1984). Repetition priming and frequency attenuation in lexical access. Journal of Experimental Psychology: Learning, Memory, and Cognition.

[CR15] Forster KI, Davis C, Schoknecht C, Carter R (1987). Masked priming with graphemically related forms: Repetition or partial activation?. Quarterly Journal of Experimental Psychology.

[CR16] Forster KI, Forster JC (2003). DMDX: A Windows display program with millisecond accuracy. Behavior Research Methods, Instruments, & Computers.

[CR17] Grainger J, Jacobs AM (1996). Orthographic processing in visual word recognition: A multiple read-out model. Psychological Review.

[CR18] Grainger J, Van Heuven WJB, Bonin P (2004). Modeling letter position coding in printed word perception. Mental lexicon: Some words to talk about words.

[CR19] Jaeger TF (2008). Categorical data analysis: Away from ANOVAs (transformation or not) and towards logit mixed models. Journal of Memory and Language.

[CR20] Kinoshita S, Kaplan L (2008). Priming of abstract letter identities in the letter match task. Quarterly Journal of Experimental Psychology.

[CR21] Kinoshita S, Lagoutaris S (2010). Priming by NUMB3R5 does not involve top-down feedback. Journal of Experimental Psychology: Learning, Memory, and Cognition.

[CR22] Kinoshita S, Norris D (2011). Does the familiarity bias hypothesis explain why there is masked priming for “NO” decisions?. Memory & Cognition.

[CR23] Kučera H, Francis WN (1967). Computational analysis of present-day American English.

[CR24] Massol S, Duñabeitia JA, Carreiras M, Grainger J (2013). Evidence for letter-specific position coding mechanisms. PLoS ONE.

[CR25] McClelland JL, Rumelhart DE (1981). An interactive activation model of context effects in letter perception: I. An account of basic findings. Psychological Review.

[CR26] Mueller ST, Weidemann CT (2012). Alphabetic letter identification: Effects of perceivability, similarity, and bias. Acta Psychologica.

[CR27] Norris D (2006). The Bayesian Reader: Explaining word recognition as an optimal Bayesian decision process. Psychological Review.

[CR28] Norris D, Kinoshita S (2008). Perception as evidence accumulation and Bayesian inference: Insights from masked priming. Journal of Experimental Psychology: General.

[CR29] Norris D, Kinoshita S (2012). Reading through a noisy channel: Why there’s nothing special about the perception of orthography. Psychological Review.

[CR30] Norris D, Kinoshita S, van Casteren M (2010). A stimulus sampling theory of letter identity and order. Journal of Memory and Language.

[CR31] Perea M, Abu Mallouh R, Carreiras M (2013). Early access to abstract representations in developing readers: Evidence from masked priming. Developmental Science.

[CR32] Perea M, Duñabeitia JA, Carreiras M (2008). R34D1NG W0RD5 W1TH NUMB3R5. Journal of Experimental Psychology: Human Perception and Performance.

[CR33] Perea M, Duñabeitia JA, Pollatsek A, Carreiras M (2009). Does the brain regularize digits and letters to the same extent?. Quarterly Journal of Experimental Psychology.

[CR34] Perea M, Panadero V (2013). Does *viotin* activate *violin* more than *viocin*? On the use of visual cues during visual-word recognition. Experimental Psychology.

[CR35] Price C, Devlin JT (2003). The myth of the visual word form area. NeuroImage.

[CR36] R Development Core Team (2013). R: A language and environment for statistical computing [Computer software].

[CR37] Vogel AC, Petersen SE, Schlaggar BL (2012). The left occipitoteporal cortex does not show preferential activity for words. Cerebral Cortex.

[CR38] Vogel EK, Woodman GF, Luck SJ (2001). Storage of features, conjunction, and objects in visual working memory. Journal of Experimental Psychology: Human Perception and Performance.

[CR39] Wang X, Yang J, Shu H, Zevin JD (2011). Left fusiform BOLD responses are inversely related to word-likeness in a one-back task. NeuroImage.

[CR40] Whitney C (2001). How the brain encodes the order of letters in a printed word: The SERIOL model and selective literature review. Psychonomic Bulletin & Review.

[CR41] Whitney C (2008). Comparison of the SERIOL and SOLAR theories of letter-position encoding. Brain and Language.

